# Phytochemical constituents and biological activities of different extracts of *Strobilanthes crispus* (L.) Bremek leaves grown in different locations of Malaysia

**DOI:** 10.1186/s12906-015-0873-3

**Published:** 2015-11-27

**Authors:** Ali Ghasemzadeh, Hawa ZE Jaafar, Asmah Rahmat

**Affiliations:** Department of Crop Science, Faculty of Agriculture, Universiti Putra Malaysia, 43400 Serdang, Selangor Malaysia; Department of Nutrition & Dietetics, Faculty of Medicine & Health Sciences, Universiti Putra Malaysia, 43400 UPM, Serdang, Selangor Malaysia

**Keywords:** *Strobilanthes crispus*, Flavonoids, Phenolic acids, UHPLC, DPPH, FRAP, Anticancer, Kelantan

## Abstract

**Background:**

*Strobilanthes crispus* is a well-known herb in Malaysia with various pharmaceutical properties. *S. crispus* is known to contain several biologically active chemical constituents which are responsible for its pharmaceutical quality.

**Methods:**

*Strobilanthes crispus* leaves grown in three different locations in Malaysia [Kelantan (North-east), Selangor (Central), and Penang (North)], were investigated for differences in the content of secondary metabolites [total phenolics content (TPC), total flavonoids content (TFC), and total saponins content (TSC)] as well as for their antioxidant and anticancer properties. Phenolic acids and flavonoids were identified using ultra-high performance liquid chromatography (UHPLC). Ferric reducing antioxidant potential (FRAP) and 1,1-diphenyl-2-picrylhydrazyl (DPPH) assays were used to evaluate the antioxidant activities. The anticancer activity of extracts against HeLa cancer cell line was evaluated using the MTT (3-(4,5-dimethylthiazol-2-yl)-2,5-diphenyltetrazolium bromide) assay.

**Results:**

Samples from the three different locations when extracted with two solvents (aqueous and ethanol extracts) yielded significantly different results for TPC, TFC, and TSC as well as for antioxidant activity. Aqueous extract of *S. crispus* leaves collected from Kelantan exhibited the highest values: TPC [12.62 mg gallic acid equivalents (GAE)/g dry matter (DM)], TFC (7.44 mg quercetin equivalents (QE)/g DM), TSC (44.7 mg diosgenin equivalents (DE)/g DM), DPPH (73.8 %), and FRAP (267.5 μM of Fe (II)/g) activity with a half-maximal inhibitory concentration (IC_50_) of 44.1 μg/mL compared to the extracts of leaves collected from the other two locations. The most important secondary metabolites identified in this study, based on concentration, were phenolics classified as followed: caffeic acid>ferulic acid>gallic acid>chlorogenic acid>trans-cinnamic acid; flavonoids: quercetin>rutin>catechin>apigenin>naringenin>kaempferol. Extracts of leaves collected from Kelantan exhibited better anticancer activity against HeLa cancer cell line with an IC_50_ of 182.5 μg/mL compared to the extracts of leaves from Selangor (IC_50_ = 266.4 μg/mL) and Penang (IC_50_ = 331.5 μg/mL) and to tamoxifen (IC_50_ = 63.4 μg/mL). *S. crispus* leaves with the highest content of secondary metabolites exhibited the most potent antioxidant and anticancer activity.

**Conclusions:**

Therefore, based on the potent antioxidant and anticancer activity of leaves extracts, it appears that *S. crispus* grown in the North-east of Malaysia (Kelantan) is a potential source of anticarcinogenic therapeutic compounds.

## Background

Herbs and natural products are important sources of medicinal compounds and their beneficial healing effects have been well recognized since ancient times. The characteristics and therapeutic effects of natural bioactive compounds, especially from plant sources including spices, have been extensively investigated. Phytochemicals are important compounds found in medicinal plants that are not essential for the normal functioning of the human body, but are active and exert beneficial effects on health or in amelioration of diseases. Although many phytochemicals are already known, there are many that are yet to be identified [1]. According to a report by the World Health Organization, 80 % of the population in developing countries depend on traditional medicine for their primary health care, and 85 % of traditional medicine is derived from plant extracts [2]. In Malaysia, herbs and spices are generally consumed raw and fresh similar to vegetables (salad), especially by the Malay community. *Strobilanthes crispus* is a well-known herb in Malaysia. This plant, locally known in Malaysia as Pecah kaca, Pecah beling, Karang jin, Bayam karang and yellow strobilanthus in English, is a plant that belongs to the Acanthacea family. It is a woody spreading shrub with glossy dark-green, opposite, elliptical-shaped leaves. *Strobilanthes crispus* is traditionally used as a folklore medicinal plant in Malaysia and Indonesia as an antidiabetic, diuretic, or antilithic agent as well as a laxative to treat constipation [3]. The plant is also known as Hei Mian Jiang Jun (Black-faced General) to the local Chinese community and the leaves of the plants are normally boiled and taken as tea or is mixed with other herbs. In addition, *S. crispus* has been reported to have various other properties including antidiabetic [4, 5], antioxidant [6, 7], antiangiogenic [8], and wound healing activities [9] as well as a hypolipidemic effect [5]. Oxidative stress is considered to be substantial, if not crucial, in the initiation and development of many current conditions and diseases, including: inflammation, autoimmune diseases, cataract, cancer, Parkinson’s disease, arteriosclerosis and aging [2, 10]. Cancer is a multi-step disease incorporating physical, environmental, metabolic, chemical and genetic factors, in which each plays a direct and/or indirect role in the induction and deterioration of cancers. Diet with high consumption of antioxidant rich fruits and vegetables reduces the risk of many cancers types, significantly suggesting that these antioxidants could be effective agents to inhibit cancer [11]. Antioxidants in the diet are very promising as cancer inhibitors because of their low toxicity, safety and general acceptance [12]. Isolated polyphenols from different plants have been considered in a number of cancer cell lines at different stages of cancer growth. For example, the isolated polyphenols from strawberry including kaempferol, quercetin, anthocyanins, coumaric acid and ellagic acid, were shown to inhibit the growth of human breast (MCF-7), oral (KB, CAL-27), colon (HT-29, HCT-116), and prostate (LNCaP, DU-145) tumor cell lines [13, 14]. The pharmaceutical activity of herbs is correlated to their content of phytochemicals. Various phytochemical groups and constituents have been identified in *S. crispus* including flavonoids, phenolic acids, alkaloids, and ester glycosides [15]. Phenolic acids identified in ethanol extracts of dried leaves of *S. crispus* included p-hydroxybenzoic acid, p-coumaric acid, caffeic acid, vanillic acid, gentinic acid, ferulic acid, and syryngic acid, and the alkaloids caffeine and tannin are also present [6]. The flavonoid compounds identified in leaves extracts of *S. crispus* included naringenin, (+)-catechin, kaempferol rutin, myricetin, apigenin, luteolin, and (−)-epicatechin [16]. Previous studies reported that environmental conditions have a substantial effect on the phytochemical content and composition [17]. Therefore, it is important to refer to the sampling location and environmental parameters when considering the content of phytochemicals and the beneficial effect on health exerted by *S. crispus*.

To the best of our knowledge, there is little information on the content and pharmaceutical quality of phenolic compounds in Malaysian *S. crispus* or whether the production of secondary metabolites differs between *S. crispus* leaves grown in different geographical locations in Malaysia. Thus, the aim of this study was to characterize the phytochemical content and investigate the antioxidant and anticancer activity in *Strobilanthes crispus* leaves grown in three different areas [North-east (Kelantan), Central (Selangor), and North (Penang)] of Malaysia.

## Methods

### Plant sampling

Samples of *S. crispus* were collected from three different area of Malaysia: Penang (North), Kelantan (North-east) and Selangor (Central) by Malaysian Agriculture Research and Development Institute (MARDI), Serdang, Selangor, Malaysia. The samples were identified by Dr Salma Idris and voucher specimens were deposited at the Herbarium of MARDI. Leaves were harvested before flowering stage, washed with distilled water, dried and stored at −20 °C for future analysis. Table 1 represent climatic and geographical information of sampling area.

**Table 1:** Climatic and geographical information of sampling area

Locations	Lowest temperature (°C)	Highest temperature (°C)	Above sea level (m)	Humidity (%)	Light intensity (μmol/m^2^/s)	Average rainfall (mm)
Penang	23	32	3	76	760	2670
Selangor	23	33	56	80	940	2600
Kelantan	21	32	42	81	1050	2500

### Extraction

#### Preparation of aqueous extract

Dried leaves (50 g) were grounded into powder followed by extraction with distilled water (1 L). Solutions were refluxed  for 2 h at 65 °C, then cooled and filtered through Whatman filter paper (No. 1) in a filter funnel, followed by evaporation under reduced pressure in an Eyela rotary evaporator to remove excess water (crude extract weight was 9.14 g).

#### Preparation of ethanol extract

Dried powdered leaves (50 g) were ground into powder followed by extraction with ethanol (1 L). Solutions were refluxed for 2 h at 65 °C. The solution was then cooled at room temperature and filtered through Whatman filter paper (No. 1), followed by evaporation under reduced pressure in an Eyela rotary evaporator to remove excess solvent. The residue was freeze-dried and dried extracts were kept at −20 °C for future analysis (crude extract weight was 2.78 g).

#### Total phenolic content

Extracts of leaves (200 μL) were diluted in 20 mL of distilled water. Folin-Ciocalteu reagent (10-fold diluted; 1 mL) was added and the mixture was incubated in total darkness for 10 min at room temperature. After this time, sodium carbonate 7.5 % (1 mL) was added and incubated for 30 min, then the absorbance of the solution was read at 765 nm using a spectrophotometer (UV2550, Shimadzu, Japan) [18]. Different concentrations of gallic acid were used to prepare a calibration curve. Results were expressed as milligram gallic acid equivalents (GAE)/g DM.

#### Total flavonoid content

Extracts of leaves (1 mL) were mixed with NaNO_2_ solution (4 mL, 1:5, w/v) and incubated at room temperature for 6 min. After this time, 0.3 mL of AlCl_3_ solution (1:10, w/v) was added, the reagents were mixed well, and the reaction was allowed to stand for another 6 min. Immediately after that, 1 M NaOH solution (2.0 mL) was added to each extract and incubated for 10 min at room temperature. The absorbance of the solutions was read at 510 nm using a spectrophotometer (UV2550, Shimadzu, Japan). Different concentrations of quercetin standard were used to prepare a calibration curve [19]. Results were expressed as milligram querectin equivalents (QE)/g DM.

#### Total saponin content

Leaves dry sample (0.5 g) was mixed with 10 mL of 80 % aqueous methanol in centrifuge tubes. The tubes were tightly capped and the contents were stirred overnight using a magnetic stirrer. The tubes were centrifuged at 3000 g for 10 min at room temperature and the supernatants were collected in 25 ml volumetric flasks. The residue was washed thrice with 5 ml of 80 % aqueous methanol. Aliquots of the samples from the flasks were used for saponin determination. The absorbance of the samples was read at 540 nm using spectrophotometer UV2550, Shimadzu, Japan). Methanol was used as a blank. Diosgenin as a standard was dissolved in methanol with varied concentrations (0.06, 0.12, 0.24, and used for the calibration curve. Results were expressed as milligram diosgenin equivalents (DE)/g DM.

#### Separation and analysis of flavonoids and phenolic acids

Ultra-high performance liquid chromatography (UHPLC, 1290 Infinity Quaternary LC System, Agilent, Santa Clara, CA, USA) was used to separate and identify the phenolics and flavonoids. The chromatographic system conditions were set as follows: mobile phase, 0.03 M orthophosphoric acid (A) and methanol HPLC grade (B); detector, UV 360 nm; column, C18 column (5.0 μm, 4.6 mm inner diameter [ID] × 250 mm); column oven temperature, 35 °C; and flow rate, 1.0 mL/min. Gradient elution was performed as follows: 0–10 min, 10 % B; 10–15 min, 50 % B; 15–20 min, 100 % B; and finally 5 min for washing. Linear regression equations were calculated using Y = aX ± b, where X is the concentration of the related compound and Y the peak area of the compound obtained from UHPLC [19]. The linearity was established by the coefficient of determination (R^2^). All flavonoids (rutin hydrate ≥94.0 %; quercetin ≥95.0 %; kaempferol ≥97.0 %; (+) catechin ≥99.0 %; naringenin 98.0 %; apigenin ≥95.0 %) and phenolic acids (gallic acid monohydrate ≥99 %; ferulic acid ≥99 %; trans-Cinnamic acid ≥99 %; chlorogenic acid ≥95 % and caffeic acid >98 %) standards were purchased from Sigma-Aldrich, Malaysia.

### *In vitro* evaluation of antioxidant activity

#### 1,1-Diphenyl-2-picrylhydrazyl (DPPH) assay

The DPPH assay was used in order to evaluate the free radical scavenging activity of *S. crispus* extracts. DPPH was dissolved in methanol at a concentration of 100 μM. The DPPH solution (3 mL) was mixed with 3 mL of various concentrations (10, 20, 40, 80 and 160 μg/mL) of *S. crispus* extracts and incubated in a dark room for 20 min at 27 °C. After incubation, the absorbance of the samples was read at 517 nm using a spectrophotometer (UV2550, Shimadzu, Japan). Butylated hydroxytoluene (BHT) and α-tocopherol were used as a positive control [20]. DPPH, BHT (≥99.0 %) and α-tocopherol (≥95.5 %) were purchase from Sigma-Aldrich, Malaysia.

The scavenging activity was calculated using the following formula:

1 $$ \%\kern0.5em \mathrm{inhibition}=\left[\left({\mathrm{absorbance}}_{\mathrm{control}}\hbox{--} {\mathrm{absorbance}}_{\mathrm{sample}}\right)/{\mathrm{absorbance}}_{\mathrm{control}}\right)\Big]\times 100 $$

#### Ferric reducing antioxidant potential (FRAP) assay

The stock solutions consisted of 10 volume of 300 mM acetate buffer (PH = 3.6), 1 volume of 10 mM TPTZ (2,4,6-tripyridyl-S-triazine) solution in 40 mM HCl, and I volume of 20 mM FeCl_3_ solution. Acetate buffer (25 mL) and TPTZ (2.5 mL) were mixed (FRAP solution), and 2.5 mL FeCl_3_ added. Leaves extract (100 μL) and deionized water (300 μL) was added to 3 mL of the FRAP solution and incubated for 30 min at 37 °C in the dark water bath. The bsorbance of the resultant solution was measured at 593 nm using a spectrophotometer (U-2001, Hitachi Instruments Inc., Tokyo, Japan) acetate buffer was used as a blank reading. A standard curve was prepared using various concentrations of FeSO_4_ × 7H_2_O. The difference between sample absorbance and blank absorbance was calculated and used to calculate the FRAP value [21].

### Determination of anticancer activity

#### Cell culture and treatment

Human cervical carcinoma cell line (HeLa cells) and normal human mammary epithelial cells were purchased from the laboratory of Molecular Biomedicie, Institute Bio-sience, University Putra Malaysia, Serdang, Selangor, Malaysia. Cells were cultured in RPMI 1640 media containing 10 % fetal bovine serum (FBS). Cell lines were incubated overnight at 37 °C in 5 % CO_2_ for cell attachment. The cells were maintained by sub-culturing in 25 cm^2^ tissue culture flasks. Cells growing in the exponential phase were used for cell viability assay.

#### MTT (3-(4,5-Dimethylthiazol-2-yl)-2,5-diphenyltetrazolium bromide) assay

The assay was conducted as follows: Cancer cells were seeded in 96-well plates at a density of 1 × 10^4^ cells/well in 100 μL RPMI. After 24 h, the medium was removed and the cells were incubated for 3 days with RPMI in the presence or absence of various concentrations of *S. crispus* leaves extract (test extracts were prepared in 0.1 % Dimethyl sulfoxide and serially diluted with media to obtain appropriate concentrations). The following concentrations of extracts were used: 20, 40, 80, 160, 320, and 640 μg/mL. Cells in the control group received only media containing 0.1 % Dimethyl sulfoxide (DMSO). After incubation, the test compound containing media was removed and washed with 200 μL of PBS followed by addition of 20 μL of MTT reagent (5 mg/mL MTT in PBS) and incubated for 4 h at 37 °C. The medium was removed and 100 μL DMSO was added and the absorbance measured using a micro plate reader at 540 nm followed by the calculation of percentage viability. 0.1 % (v/v) DMSO in medium was used as negative control. The cell viability was determined using the formula:

2 $$ \mathrm{Viability}\kern0.1em \left(\%\right)=100-\left(\mathrm{optical}\kern0.1em \mathrm{density}\kern0.1em \mathrm{of}\kern0.1em \mathrm{sample}/\mathrm{optical}\kern0.1em \mathrm{density}\kern0.1em \mathrm{of}\kern0.1em \mathrm{control}\right)\times 100 $$

Optical density of sample = absorbance of cells treated with extract - absorbance of cells treated with 0.1% DMSO medium. Optical density of control: absorbance of cells treated with 0.1% DMSO medium. Each point represents the mean of triplicate experiments.

## Results and discussion

### Total phenolic content (TPC) and total flavonoid content (TFC)

Aqueous and ethanol extracts of *S. crispus* leaves collected from three different locations in Malaysia, were evaluated for phytochemical composition. As shown in Table 2, the leaves collected from different locations had significantly different concentrations of TPC were also dependent on the solvent. Aqueous extracts from the leaves collected from Kelantan exhibited the highest level of TPC (12.62 mg GAE/g DM) compared to that of aqueous extracts of leaves collected from Selangor (10.45 mg GAE/g DM) and Penang (9.12 mg GAE/g DM). Extraction with water rather than ethanol enhanced the levels of TPC by about 37.3 % in extracts of leaves collected from Penang, 49.2 % in extracts of leaves from Selangor, and 42.4 % in extracts of leaves from Kelanton. The extracts from the *S. crispus* leaves had a higher TPC than that reported previously for other herbs including *Marrubium vulgare* (3.86 mg/100 g DM), *Rosmarinus officinalis* (1.71 mg/100 g DM), *Artemisia vulgaris* (3.83 mg/100 g DM), *Levisticum officinale* (0.72 mg/100 g DM), *Epilobium hirsutum* (4.03 mg/100 g DM), and *Chelidonium majus* (2.09 mg/100 g DM) [22].

**Table 2:** Total phenolic, total flavonoid and total saponin content of *S. crispus* leaves, extracted with different solvent and collected from three different locations

Sampling location	Solvent	TPC (mg GAE/g DM)	TFC (mg QE/g DM)	TSC (mg DE/g DM)
Penang	aqueous	9.12 ± 0.726^c^	5.15 ± 0.345^c^	26.2 ± 2.451^e^
	ethanol	6.64 ± 0.668^e^	3.28 ± 0.266^e^	22.5 ± 2.328^f^
Selangor	aqueous	10.45 ± 0.689^b^	6.20 ± 0.322^b^	34.4 ± 2.336^c^
	ethanol	7.00 ± 0.566^e^	3.58 ± 0.291^e^	30.4 ± 2.842^d^
Kelantan	aqueous	12.62 ± 0.512^a^	7.44 ± 0.429^a^	44.7 ± 3.726^a^
	ethanol	8.86 ± 0.829^d^	4.66 ± 0.284^d^	38.8 ± 2.458^b^

All analyses are the mean of triplicate measurements ± standard deviation. Means not sharing a common letter in each column were significantly different at *P* < 0.05

The amount of TFC was between 3.28 and 7.44 mg QE/g DM and, the different locations and solvents significantly influenced the TFC. Leaves extract of *S. crispus* obtained from Kelantan exhibited highest TFC (44.0 mg QE/g DM) followed by Selangor (6.20 mg QE/g DM) and Penang (5.15 mg QE/g DM) samples. Similar to TPC, compared to ethanol extraction, aqueous extraction enhanced the level of TFC by about 57 % (Penang), 73.1 % (Selangor), and 59.6 % (Kelantan). It is apparent from Table 2 that the solubility of polyphenolic compounds is higher in aqueous solvents than that in ethanol. The TFC of extracts of *S. crispus* leaves from Kelantan was higher than that previously reported for herbs including *Cymbopogon citratus* (3.05 mg/g DM), *Mentha piperita* (3.01 mg/g DM), *Citrus bergamia* (2.11 mg/g DM), *Mentha piperita* (3.16 mg/g DM) and Jasminum (3.05 mg/g DM) [23].

Herbs may contain saponins, which are important phytochemicals with a wide range of medicinal properties, including anticarcinogenic, anti-inflammatory, antioxidant, and antimicrobial activities [24]. A variable saponin content was identified in different herbs and plants including onion, garlic [25], Kacip Fatimah [26], soya [27], peas [28] and notoginseng [29]. In the current study, *S. crispus* leaves from all locations had high TSC. Aqueous extracts of *S. crispus* leaves from Kelantan had the highest TSC (44.7 mg DE/g DM) followed by Selangor (34.4 mg DE/g DM) and Penang samples (26.2 mg DE/g DM). The TSC increased by about 16.4 % (Penang), 13.15 % (Selangor), and 15.2 % (Kelantan) when an aqueous solvent was used rather than ethanol.

### Antioxidant activity

**Fig. 1:**
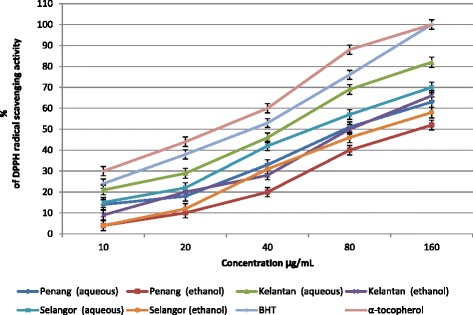
The DPPH radical scavenging activity of *S. crispus* leaves, extracted with different solvents and collected from three different locations. *Bars* represent standard error of the means

The antioxidant properties of *S. crispus* leaves extracts from three different location of Malaysia were determined using two different methods namely DPPH and FRAP assays. The results from both assays showed significant differences in the antioxidant activity owing to different sampling locations and solvent type (Table 3) with aqueous extracts having greater DPPH free radical scavenging activity and ferric reducing antioxidant potential (FRAP) than that of the ethanol extracts. At a concentration of 100 μg/mL, the highest DPPH free radical scavenging activity was observed in the aqueous extract of *S. crispus* leaves from Kelantan (73.8 %) followed by Selangor (62.4 %) and Penang (54.6 %), with the half maximal inhibitory concentration (IC_50_) of 44.1, 58.2, and 78.3 μg/mL, respectively compared to BHT (37.5 μg/mL) and α-tocopherol (26.4 μg/mL), (Fig. 1). It should be noted that a lower IC_50_ value represents a better free radical inhibition (strong free radical inhibitors are active at low concentrations). Thus, the results indicated that aqueous extracts have higher antioxidant activity compared to ethanolic extracts. The FRAP value was in the range of 59.8–267.5 μM of Fe (II)/g with the highest and lowest reducing activity observed in the aqueous extracts from Kelantan leaves and ethanol extracts from Penang leaves, respectively. The FRAP activity increased by about 96.6 % (Penang), 42.5 % (Selangor), and 32.5 % (Kelantan) when extraction was performed with aqueous solvent rather than ethanol. Qader et al. [7] reported that aqueous extracts of *S. crispus* leaves (1 mg/mL) showed antioxidant activity with Fe^2+^ reducing ability (1182 mM/g) compared to gallic acid (1216.67 mmol/g) using the FRAP assay. In a previous study, ethanol extract of *S.crispus* leaves (0.2 %) showed antioxidant activity with Fe^2+^ reducing ability 180 % compared to vitamin E (78 %) using ferric reducing antioxidant potential [6]. Muslim et al. [8] reported that aqueous extract (800 μg/mL) of *S.crispus* leaves exhibited 17.46 % scavenging activity. Bakar et al. [30] compared the antioxidant activity of *S.crispus* (unfermented tea) with green and black tea using FRAP and DPPH method and showed that *S.crispus* exhibited highest FRAP value (2091 μmol/L) and lowest DPPH free radical scavenging activity (63.21 %) compared to green (FRAP: 56.7 μmol/L; DPPH: 79.56 %) and black tea (FRAP: 34.3 μmol/L: DPPH: 74.27 %). Several studies reported a significant correlation between the antioxidant activity of herbs and the phytochemical content [19, 22, 31, 32]. In the current study, aqueous extracts of *S. crispus* leaves collected from Kelantan had the highest content of total flavonoids, total phenolics, and total saponins together with high antioxidant properties.

**Table 3:** Antioxidant activity of *S. crispus* leaves, extracted with different solvent and collected from three different locations

Sampling location	Solvent	DPPH free radical scavenging activity (%)	IC_50_ (μg/mL)	Ferric reducing antioxidant potential (μM of Fe (II)/g)	IC_50_ (μg/mL)
Penang	aqueous	54.6 ± 2.776^c^	78.3 ± 2.64^c^	117.6 ± 4.305^g^	80.6 ± 3.47^c^
	ethanol	41.7 ± 3.261^f^	146.5 ± 3.60^a^	59.8 ± 4.026^h^	148.3 ± 3.48^a^
Selangor	aqueous	62.4 ± 2.226^b^	58.2 ± 2.01^d^	180.6 ± 6.208^e^	63.2 ± 2.16^d^
	ethanol	49.2 ± 1.894^e^	117.6 ± 2.64^b^	126.7 ± 4.550^f^	122.6 ± 2.77^b^
Kelantan	aqueous	73.8 ± 3.385^a^	44.1 ± 3.16^e^	267.5 ± 9.568^b^	52.8 ± 1.76^e^
	ethanol	55.4 ± 2.628^c^	80.5 ± 3.28^c^	201.8 ± 7.452^d^	80.7 ± 3.04^c^
BHT		51.6 ± 3.441^d^	37.5 ± 1.59^f^	250.6 ± 7.255^c^	40.7 ± 1.15^f^
α-tocopherol		60.2 ± 4.266^b^	26.4 ± 1.24^g^	322.1 ± 10.150^a^	29.1 ± 1.52^g^

All analyses are the mean of triplicate measurements ± standard deviation. Means not sharing a common letter in each column were significantly different at *p* < 0.05

### Separation and identification of phenolic acids and flavonoids

In the current study, five phenolic acids (gallic acid, ferulic acid, cinnamic acid, chlorogenic acid, and caffeic acid) and six flavonoid compounds (quercetin, rutin, kaempferol, catechin, naringenin, and apigenin) were identified from the extracts of *S. crispus* leaves collected from three different locations (Table 4). The aqueous rather than the ethanolic extraction method was chosen for profiling of phenolic acids and flavonoids in order to maximize the TPC, TFC, TSC, and antioxidant activity. The results from the three different sampling locations showed significant differences. The highest content of caffeic acid (2.95 mg/g DM), ferulic acid (1.76 mg/g DM), chlorogenic acid (0.75 mg/g DM), and gallic acid (1.45 mg/g DM) was observed in extracts of leaves collected from Kelantan. trans-Cinnamic acid at a concentration of 0.44 mg/g DM was detected in extracts of leaves from Penang but not in the extracts of the leaves from the other two locations. Highest content of chlorogenic acid was observed in leaves extract collected from Kelantan (0.75 mg/g DM) followed by Selangor (0.5 mg/g DM). Chlorogenic acid was not detected in the extracts of leaves from Penang. The most important phenolic acids identified in this study, based on concentration were caffeic acid > ferulic acid > gallic acid > chlorogenic acid > trans-cinnamic acid. The flavonoids compounds were significantly different between the extracts of leaves from the three different locations. Compared to the extracts of leaves from the other two locations, extracts of *S. crispus* leaves collected from Kelantan had the highest content of quercetin (1.95 mg/g DM), rutin (1.48 mg/g DM), kaempferol (0.56 mg/g DM), catechin (1.12 mg/g DM), naringenin (0.58 mg/g DM), and apigenin (0.0.92 mg/g DM). Kaempferol and naringenin were not detected in the extracts of leaves from Penang, and naringenin was not detected in the extracts of leaves collected from Selangor. Quercetin has been reported to be a potent antioxidant with anticancer activity [33, 34]. *S. crispus* leaves had high levels of quercetin compared to that in the other herbs such as *Salvia officinalis* (1.78 mg/g DM), *Silybum marianum* (0.23 mg/g DM), *Archangelica officinalis* (0.48 mg/g DM), *Hypericum perforatum* (0.49 mg/g DM), *Syzygium aromaticum* (1.55 mg/g DM), but lower quecetin content than *Chelidonium majus* (7.59 mg/g DM), *Epilobium hirsutum* (2.14 mg/g DM), and *Juglans regia* (4.60 mg/g DM) [22]. In addition, rutin and catechin which have been reported to have anti- and pro-oxidative activity [35, 36] were detected in *S. crispus* leaves extracts in quantities higher (particularly in the Kelantan sample) than that reported for a number of other herbs and crops such as Buckwheat (rutin: 0.127 mg/g DM; catechin: 0.033 mg/g DM) [37], Pandan (rutin: 0.082 mg/g DM; catechin: 0.527 mg/g DM) [38], and Curry leaf (rutin: 0.042 mg/g DM; catechin: 0.325 mg/g DM) [39]. The highest kaempferol (0.56 mg/g DM) and naringenin (0.92 mg/g DM) content was identified in the extracts of *S. crispus* leaves collected from Kelantan. The most important flavonoids identified in this study, based on concentration were quercetin > rutin > catechin > apigenin > naringenin > kaempferol.

**Table 4:** Identified of phenolic acids and flavonoids from *S. crispus* leaves collected from three different locations

Secondary metabolites	Penang	Selangor	Kelantan
Phenolic acids			
Caffeic acid	1.94 ± 0.566^c^	2.52 ± 0.206^b^	2.95 ± 0.311^a^
Ferulic acid	0.85 ± 0.426^c^	1.15 ± 0.112^b^	1.76 ± 0.160^a^
trans-Cinnamic acid	0.44 ± 0.335^a^	ND	ND
Chlorogenic acid	ND	0.5 ± 0.116^b^	0.75 ± 0.128^a^
Gallic acid	0.8 ± 0.228^c^	1.08 ± 0.221^b^	1.45 ± 0.326^a^
Flavonoids			
Quercetin	0.82 ± 0.346^c^	1.4 ± 0.330^b^	1.95 ± 0.334^a^
Rutin	0.79 ± 0.122^c^	1.04 ± 0.166^b^	1.48 ± 0.269^a^
Kaempferol	ND	0.54 ± 0.066^a^	0.56 ± 0.041^a^
Catechin	0.65 ± 0.244^b^	0.72 ± 0.114^b^	1.12 ± 0.220^a^
Naringenin	ND	ND	0.58 ± 0.063^a^
Apigenin	0.33 ± 0.106^c^	0.6 ± 0.105^b^	0.92 ± 0.325^a^

All analyses are the mean of triplicate measurements ± standard deviation. Results expressed in mg/g DM. Means not sharing a common letter in each row were significantly different at *p* < 0.05. *ND* represent not detected

Comparing the three different sampling locations, the concentration of polyphenols decreased in the following order: Kelantan>Selangor>Penang. This variation in the content of phenolic acids and flavonoids in *S. crispus* leaves could be related to the differences in the weather conditions or soil nutrition and type, which have been reported previously [40–42]. The height above sea level and light intensity differs between these three sampling locations (Table 1). Light has been shown to be the most important environmental factor influencing anthocyanin biosynthesis in plants [43, 44]. High light intensity has been shown to enhance the synthesis and production of polyphenols in different plants [44–46]. The results of this research support the hypothesis that differences in phenolic acids and flavonoid synthesis could be related to increasing light intensity at the three different locations (from North-east to North). Increasing light intensity increases net photosynthesis and exceeds the carbon, which leads to an increase in flavonoids and phenolic production in the plants [47]. According to the “overflow metabolism” concept, when carbon production exceeds the carbon demand associated with plant growth, the excess carbon is channeled into biosynthesis of secondary metabolites [48]. Also, high light intensity, induces phenylalanine ammonia-lyase enzyme activity which is a key enzyme for phenolics and flavonoid synthesis in plants [43]. A recent study by Wang et al. [44] also reported that light stimulated the production of phenolic acids and flavonoids. In a further major study, Jaakola et al. [49], found that the production of flavonoid compounds in bilberry leaves was enhanced by increasing light intensity. Light increases the biosynthesis of polyphenolics in plants by increasing the activity of phenylalanine ammonia-lyase, which is a key enzyme in the shikimic acid pathway converting phenylalanine into coumaric acid. Coumaric acid is the initial precursor molecule involved in the synthesis of phenolic components in plants [50].

### Anticancer activity

**Fig. 2:**
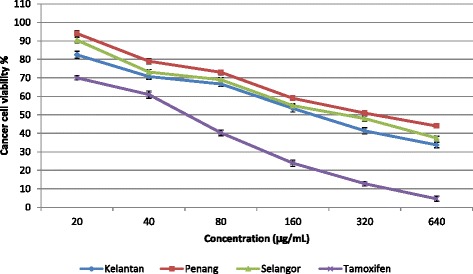
Anticancer activity of *S. crispus* leaves extracts (collected from three different locations) against HeLa cancer cell line. *Bars* represent standard error of the means

**Fig. 3:**
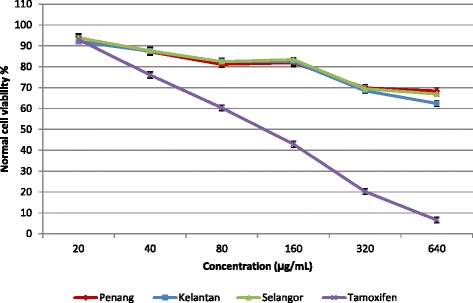
Toxicity effect of *S. crispus* leaves extracts (collected from three different locations) against normal cell line. *Bars* represent standard error of the means

Aqueous extracts of *S. crispus* leaves (20–640 μg/mL) collected from three different locations (Penang, Selangor, and Kelantan) were tested for anticancer activity against the HeLa cells (Fig. 2). Significant differences (*p* < 0.05) between IC_50_ value of different location was observed. Extracts of leaves from Kelantan exhibited potent anticancer activity with IC_50_ of 182.5 μg/mL compared to that of extracts of Selangor (IC_50_ = 266.4 μg/mL) and Penang samples (IC_50_ = 331.5 μg/mL) and compared to tamoxifen (IC_50_ = 63.4 μg/mL). No toxicity was observed against normal cells at concentrations of 20–640 μg/mL although tamoxifen was cytotoxic against the normal cell line at concentrations above 127.4 μg/mL (Fig. 3). Our finding in this research are in accordance with those of Hanisa et al. [51] who reported that aqueous extracts of *S. crispus* at concentrations of <200 μg/mL, were not cytotoxic for BHK (baby hamster kidney), VERO (kidney epithelial cells), or RK (rabbit kidney) cell lines. Previous reports have described the anticancer activity of *S. crispus* leaves against different cancer cell lines. Previously, it was reported that aqueous extracts of *S. crispus* leaves (25 mg/mL) were cytotoxic for the liver hepatocellular carcinoma cell line (Hep G2), colon carcinoma cell line (HCT116), ductal breast epithelial tumor cell line (T-47D), lung cancer cell Line (NCI-H23), and breast cancer cell line (MCF-7) with IC_50_ values of >200, >200, >200, >200, and 120.7 μg/mL, respectively [8]. Extracts of *S. crispus* leaves (0–100 μg/mL) showed cytotoxic activity against Hela, colon adenocarcinoma cells (HT-29), and breast cancer cells (MDA-MB-231 and MCF-7) with IC_50_ values of 78, 52, >100, and 30 μg/mL respectively [52]. In other study, Cheng reported that various extract of *S. crispus* leaves exhibited cytotoxicities against MCF-7, DU 145 and HT-3 cell lines but, IC_50_ values of most of the extracts were not achievable [53]. Chong et al. showed that hexan extract of S. crispus induced apoptosis via enhanced caspase-3/7 activation in HeLa cancer cell line [54]. The anticancer properties of herbs and spice is directly related directly to their phytochemical content [55]. In the current study, the *S. crispus* leaves with the highest content of secondary metabolites exhibited the most potent antioxidant and anticancer activity. In general, therefore, it appears that the potent antioxidant and anticancer activity of *S. crispus* grown in the North-east of Malaysia may be attributed to the high level of phytochemicals.

## Conclusion

This study demonstrated that aqueous solvent rather than ethanol is recommended for extraction of secondary metabolites from *S. crispus* leaves. The levels of secondary metabolites and the pharmaceutical quality of *S. crispus* leaves decreased in the leaves from the North-east (Kelantan) to North (Penang) of Malaysia in the following order: Kelantan>Selangor>Penang. The extracts from *S. crispus* leaves exhibited a promising anticancer activity against the HeLa cancer cell line. The extracts contained substantial amounts of effective phenolic compounds such as caffeic acid, quercetin, rutin, and catechin, which can inhibit the growth of HeLa cancer cells. These findings indicate that *S. crispus* grown in the North-east of Malaysia (Kelantan) is a potential source of anticarcinogenic therapeutic compounds and suggest areas for further investigation. These findings suggested that *S. crispus* that acted as apoptotic inducer could become a potential anticancer agent in pharmaceutical development. This research has thrown up many questions in need of further investigation.

## Abbreviations

BHT: Butylated hydroxytoluene
DM: Dry Material
DPPH: 1,1-diphenyl-2-picrylhydrazyl radical
FRAP: Ferric Reducing Antioxidant Potential
IC_50_: Half-maximal inhibitory concentration
MARDI: Malaysian Agriculture Research and Development Institute
MTT: 3-(4,5-dimethylthiazol-2-yl)-2,5-diphenyltetrazolium bromide
TPC: Total Phenolics Content
TFC: Total Flavonoids Content
TSC: Total Saponins Content
TPTZ: 2,4,6-tripyridyl-S-triazine
UHPLC: Ultra-High Performance Liquid Chromatography

## References

[1] Boyer J, Liu RH (2004). Apple phytochemicals and their health benefits. Nutr J.

[2] World Health Organization (2002). WHO traditional medicine strategy 2002–2005.

[3] Menant M (1980). Medicinal plants of east and south east Asia.

[4] Norfarizan-Hanoon N, Asmah R, Rokiah M, Fauziah O, Faridah H (2009). Antihyperglycemic, hypolipidemic and antioxidant enzymes effect of *Strobilanthes crispus* juice in normal and streptozotocin-induced diabetic male and female rats. Int J Pharmacol.

[5] Fadzelly AM, Asmah R, Fauziah O (2006). Effects of *Strobilanthes crispus* tea aqueous extracts on glucose and lipid profile in normal and streptozotocin-induced hyperglycemic rats. Plant Foods Hum Nutr.

[6] Ismail M, Manickam E, Danial AM, Rahmat A, Yahaya A (2000). Chemical composition and antioxidant activity of *Strobilanthes crispus* leaf extract. J Nutr Biochem.

[7] Qader SW, Abdulla MA, Chua LS, Najim N, Zain MM, Hamdan S (2011). Antioxidant, total phenolic content and cytotoxicity evaluation of selected Malaysian plants. Molecules.

[8] Muslim N, Ng K, Itam A, Nassar Z, Ismail Z, Majid A (2010). Evaluation of cytotoxic, anti-angiogenic and antioxidant properties of standardized extracts of *Strobilanthes crispus* leaves. Int J Pharmacol.

[9] Al-Henhena N, Mahmood A, Al-Magrami A, Nor Syuhada A, Zahra A, Summaya M (2011). Histological study of wound healing potential by ethanol leaf extract of *Strobilanthes crispus* in rats. J Med Plants Res.

[10] Black PH, Garbutt LD (2002). Stress, inflammation and cardiovascular disease. J Psychosom Res.

[11] Fimognari C, Berti F, Nüsse M, Cantelli Forti G, Hrelia P (2005). *In vitro* antitumor activity of cyanidin-3-O-β-glucopyranoside. Chemotherapy.

[12] Fresco P, Borges F, Diniz C, Marques M (2006). New insights on the anticancer properties of dietary polyphenols. Med Res Rev.

[13] Zhang Y, Seeram NP, Lee R, Feng L, Heber D (2008). Isolation and identification of strawberry phenolics with antioxidant and human cancer cell antiproliferative properties. J Agric Food Chem.

[14] Damianaki A, Bakogeorgou E, Kampa M, Notas G, Hatzoglou A, Panagiotou S (2000). Potent inhibitory action of red wine polyphenols on human breast cancer cells. J Cell Biochem.

[15] Nurraihana H, Norfarizan-Hanoon N (2013). Phytochemistry, pharmacology and toxicology properties of *Strobilanthes crispus*. Int Food Res J.

[16] Liza M, Rahman RA, Mandana B, Jinap S, Rahmat A, Zaidul I (2010). Supercritical carbon dioxide extraction of bioactive flavonoid from *Strobilanthes crispus* (Pecah Kaca). Food Bioprod Process.

[17] Yaniv Z, Dudai N (2014). Medicinal and aromatic plants of the Middle-East, vol. 2: Springer.

[18] Jayaprakasha G, Patil BS (2007). *In vitro* evaluation of the antioxidant activities in fruit extracts from citron and blood orange. Food Chem.

[19] Ghasemzadeh A, Nasiri A, Jaafar HZ, Baghdadi A, Ahmad I (2014). Changes in phytochemical synthesis, chalcone synthase activity and pharmaceutical qualities of Sabah snake grass (*Clinacanthus nutans* L.) in relation to plant age. Molecules.

[20] Singh R, Chidambara Murthy K, Jayaprakasha G (2002). Studies on the antioxidant activity of pomegranate (*Punica granatum*) peel and seed extracts using *in vitro* models. J Agric Food Chem.

[21] Dudonne S, Vitrac X, Coutiere P, Woillez M, Mérillon J-M (2009). Comparative study of antioxidant properties and total phenolic content of 30 plant extracts of industrial interest using DPPH, ABTS, FRAP, SOD, and ORAC assays. J Agric Food Chem.

[22] Wojdyło A, Oszmiański J, Czemerys R (2007). Antioxidant activity and phenolic compounds in 32 selected herbs. Food Chem.

[23] Yoo KM, Lee CH, Lee H, Moon B, Lee CY (2008). Relative antioxidant and cytoprotective activities of common herbs. Food Chem.

[24] Rao Α, Gurfinkel D (2000). The bioactivity of saponins: triterpenoid and steroidal glycosides. Drug Metabol Drug Interact.

[25] Lanzotti V (2006). The analysis of onion and garlic. J Chromatogr A.

[26] Karimi E, Jaafar HZ, Ahmad S (2011). Phytochemical analysis and antimicrobial activities of methanolic extracts of leaf, stem and root from different varieties of Labisa pumila Benth. Molecules.

[27] Ireland PA, Dziedzic SZ, Kearsley MW (1986). Saponin content of soya and some commercial soya products by means of high‐performance liquid chromatography of the sapogenins. J Sci Food Agric.

[28] Heng L, Vincken JP, van Koningsveld G, Legger A, Gruppen H, van Boekel T (2006). Bitterness of saponins and their content in dry peas. J Sci Food Agric.

[29] Lau A-J, Woo S-O, Koh H-L (2003). Analysis of saponins in raw and steamed Panax notoginseng using high-performance liquid chromatography with diode array detection. J Chromatogr A.

[30] Bakar MFA, Teh AH, Rahmat A, Hashim N, Othman F, Fakurazi S (2006). Antiproliferative properties and antioxidant activity of various types of *Strobilanthes crispus* tea. Int J Canc Res.

[31] Heim KE, Tagliaferro AR, Bobilya DJ (2002). Flavonoid antioxidants: chemistry, metabolism and structure-activity relationships. J Nutr Biochem.

[32] Braca A, Sortino C, Politi M, Morelli I, Mendez J (2002). Antioxidant activity of flavonoids from Licania licaniaeflora. J Ethnopharmacol.

[33] Lu J, Papp LV, Fang J, Rodriguez-Nieto S, Zhivotovsky B, Holmgren A (2006). Inhibition of mammalian thioredoxin reductase by some flavonoids: implications for myricetin and quercetin anticancer activity. Cancer Res.

[34] Lee L-T, Huang Y-T, Hwang J-J, Lee P, Ke F-C, Nair MP (2001). Blockade of the epidermal growth factor receptor tyrosine kinase activity by quercetin and luteolin leads to growth inhibition and apoptosis of pancreatic tumor cells. Anticancer Res.

[35] Kessler M, Ubeaud G, Jung L (2003). Anti‐and pro‐oxidant activity of rutin and quercetin derivatives. J Pharm Pharmacol.

[36] Isemura M, Saeki K, Kimura T, Hayakawa S, Minami T, Sazuka M (2000). Tea catechins and related polyphenols as anti‐cancer agents. Biofactors.

[37] Danila A-M, Kotani A, Hakamata H, Kusu F (2007). Determination of rutin, catechin, epicatechin, and epicatechin gallate in buckwheat (*Fagopyrum esculentum* Moench) by micro-high-performance liquid chromatography with electrochemical detection. J Agric Food Chem.

[38] Ghasemzadeh A, Jaafar HZ (2013). Profiling of phenolic compounds and their antioxidant and anticancer activities in pandan (*Pandanus amaryllifolius* Roxb.) extracts from different locations of Malaysia. BMC Compl Alternative Med.

[39] Ghasemzadeh A, Jaafar HZ, Rahmat A, Devarajan T. Evaluation of bioactive compounds, pharmaceutical quality, and anticancer activity of curry leaf (*Murraya koenigii* L.). Evid base Compl Alternative Med. 2014, 2014;1-8. 10.1155/2014/873803 PMC3947808 24693327

[40] Jaakola L, Hohtola A (2010). Effect of latitude on flavonoid biosynthesis in plants. Plant Cell Environ.

[41] Bourgaud F, Gravot A, Milesi S, Gontier E (2001). Production of plant secondary metabolites: a historical perspective. Plant Sci.

[42] Mori K, Sugaya S, Gemma H (2005). Decreased anthocyanin biosynthesis in grape berries grown under elevated night temperature condition. Sci Hortic.

[43] Hahlbrock K, Wellmann E (1970). Light-induced flavone biosynthesis and activity of phenylalanine ammonia-lyase and UDP-apiose synthetase in cell suspension cultures of Petroselinum hortense. Planta.

[44] Wang SY, Chen C-T, Wang CY (2009). The influence of light and maturity on fruit quality and flavonoid content of red raspberries. Food Chem.

[45] Liu C-Z, Guo C, Wang Y-C, Ouyang F (2002). Effect of light irradiation on hairy root growth and artemisinin biosynthesis of *Artemisia annua* L. Process Biochem.

[46] Ghasemzadeh A, Jaafar HZ, Rahmat A, Wahab PEM, Halim MRA (2010). Effect of different light intensities on total phenolics and flavonoids synthesis and anti-oxidant activities in young ginger varieties (*Zingiber officinale* Roscoe). Int J Mol Sci.

[47] Karimi E, Jaafar HZ, Ghasemzadeh A, Ibrahim MH (2013). Light intensity effects on production and antioxidant activity of flavonoids and phenolic compounds in leaves, stems and roots of three varieties of *Labisia pumila* Benth. Aust J Crop Sci.

[48] Matsuki M (1996). Regulation of plant phenolic synthesis: from biochemistry to ecology and evolution. Aust J Bot.

[49] Jaakola L, Määttä-Riihinen K, Kärenlampi S, Hohtola A (2004). Activation of flavonoid biosynthesis by solar radiation in bilberry (*Vaccinium myrtillus* L.) leaves. Planta.

[50] Toor R, Savage G, Lister C (2006). Seasonal variations in the antioxidant composition of greenhouse grown tomatoes. J Food Compos Anal.

[51] Hanisa H, Mohdazmi M, Suhaila M, Hakim M: Effects of *centella asiatica* L., *curcuma longa* L., and *strobilanthes crispus* L. extracts on 3 kidney cell lines: *in vitro* cytotoxicity analysis Int J Pharm Pharmaceut Sci. 2014;6:388-392.

[52] Chong HZ, Rahmat A, Yeap SK, Akim AM, Alitheen NB, Othman F (2012). *In vitro* cytotoxicity of *Strobilanthes crispus* ethanol extract on hormone dependent human breast adenocarcinoma MCF-7 cell. BMC Compl Alternative Med.

[53] Cheng CL (2008). Pharmacological evaluation of *strobilanthes crispus* (L.) Blume.

[54] Chong YH, Koh RY, Ling APK, Chye SM, Yew MY (2014). *Strobilanthes crispus* extract induces apoptosis through enhanced caspases activities in cervical cancer cells. International Conference on Biological, Environment and Food Engineering (BEFE-2014) August 4-5, 2014; Bali, Indonesia.

[55] Liu RH (2003). Health benefits of fruit and vegetables are from additive and synergistic combinations of phytochemicals. Am J Clin Nutr.

